# Long-Term Activity Recognition from Wristwatch Accelerometer Data ^*^

**DOI:** 10.3390/s141222500

**Published:** 2014-11-27

**Authors:** Enrique Garcia-Ceja, Ramon F. Brena, Jose C. Carrasco-Jimenez, Leonardo Garrido

**Affiliations:** Tecnologico de Monterrey, Campus Monterrey, Av. Eugenio Garza Sada 2501 Sur, Monterrey 64849, Mexico; E-Mails: ramon.brena@itesm.mx (R.F.B.); jccarrasco05@gmail.com (J.C.C.-J.); leonardo.garrido@itesm.mx (L.G.)

**Keywords:** activity recognition, long-term activities, accelerometer sensor, CRF, HMM, Viterbi, clustering, subclassing, watch, context-aware

## Abstract

With the development of wearable devices that have several embedded sensors, it is possible to collect data that can be analyzed in order to understand the user's needs and provide personalized services. Examples of these types of devices are smartphones, fitness-bracelets, smartwatches, just to mention a few. In the last years, several works have used these devices to recognize simple activities like running, walking, sleeping, and other physical activities. There has also been research on recognizing complex activities like cooking, sporting, and taking medication, but these generally require the installation of external sensors that may become obtrusive to the user. In this work we used acceleration data from a wristwatch in order to identify long-term activities. We compare the use of Hidden Markov Models and Conditional Random Fields for the segmentation task. We also added prior knowledge into the models regarding the duration of the activities by coding them as constraints and sequence patterns were added in the form of feature functions. We also performed subclassing in order to deal with the problem of intra-class fragmentation, which arises when the same label is applied to activities that are conceptually the same but very different from the acceleration point of view.

## 1. Introduction

Human activity recognition is an important task for ambient intelligent systems. Being able to recognize the state of a person can provide us with valuable information that can be used as input for other systems. For example, in healthcare, fall detection can be used to alert the medical staff in case of an accident; in personal assistant applications, the current activity could be used to improve the recommendations and reminders. For example, in [1] a framework for activity inference is proposed, which is based on the idea that it is possible to classify activities from the handled artifacts used to perform each activity. They presented a practical case in a nursing home to characterize the activities that caregivers perform in providing healthcare of elders with restricted mobility. Han *et al.* [2] proposed a healthcare framework to manage lifestyle diseases by monitoring long-term activities and reporting irregular and unhealthy patterns to a doctor and a caregiver.

With the development of wearable devices that have several embedded sensors, it has become possible to collect different types of data that can be analyzed in order to understand the user's needs and provide personalized services. Examples of these types of devices are smartphones, fitness bracelets [3], smartwatches [4], *etc*. With the miniaturization of sensors, it is now possible to collect data about acceleration, humidity, rotation, position, magnetic field, and light intensity with small wearable devices. In recent years, simple human activity recognition has been achieved successfully; however, complex activity recognition is still challenging and remains an active area of research. Generally, simple activities do not depend on the context, *i.e.*, they can exist by themselves and they last for only a few seconds. Examples of this type of activities are: running, walking, resting, sitting, *etc*. More complex and long-term activities are composed of a collection of simple activities and may include additional contextual information like time of the day, spatial location, interactions with other people and objects. Examples of this type of activities include: cooking, sporting, commuting, taking medication, among other types of activities. The recognition of these activities generally requires more sensors and a fixed infrastructure (e.g., video cameras, RFID tags, several accelerometers, magnetic sensors). In this work we focus on the problem of recognizing sequential long-term activities from wristwatch accelerometer data and the problem can be stated as follows: Given a sequence of accelerometer data recorded from a wristwatch, the task is to recognize what long-term activities were performed by the user and the order in which they occurred, *i.e.*, their segmentation. In this work, we focused on activities of daily living, such as shopping, exercising, working, taking lunch, *etc.*, since they are useful to living independently [5]. This information could also be used by health and wellbeing applications such as Bewell [6] in order to assist individuals in maintaining a healthy lifestyle. Another potential application is that the activities could be further characterized in order to understand their quality. For example, when doing exercise, we may want to know if the user is guarding against injuries or performing the activity with confidence. Aung *et al.* [7] and Singh *et al.* [8] have discussed the importance of detecting the quality of activities to provide a more tailored support or feedback in mental and physical rehabilitation.

To be able to recognize the long-term activities, first we decomposed the accelerometer data into a sequence of *primitives* [9]; thus, each long-term activity is represented as a string where each symbol is a *primitive*. This process is called *vector quantization* ([10], pp. 122–131) and will be described in Section 5. A *primitive*, conceptually represents a simple activity, e.g., running, walking, *etc.*, but there is not necessarily a one-to-one mapping between the primitives and activities that exist in our “human vocabulary”. These *primitives* are automatically discovered from the data [11]. The rationale for extracting these *primitives* is that the data can be represented as a sequence of characters, and thus, sequence patterns specific to each of the activities can be looked for in order to increase the discrimination power between different activities. This type of representation is also suitable to perform activity prediction [12] (which will be left as future work). We used the Viterbi algorithm [13,14] on a Hidden Markov Model (HMM) ([10], Chapter 6) and on a Conditional Random Field (CRF) [15] to perform the segmentation and compared the results.

Within the HMMs and CRFs context, the sequence of *primitives* can be seen as the sequence of observations. For each observation we want to know the class (the activity) that generated it. The Viterbi algorithm is a dynamic programming method to find the best state sequence (in this case, the sequence of activities) that generated the observations (*primitives*) according to some optimality criterion. We used a modified version of this algorithm to add a constraint that takes into account the activities minimum lifespan. For example, if we know that the *shopping* activity takes at least 10 min, we can rule out state sequences that are shorter than 10 min. In our previous work of activity segmentation with an HMM, we called this a k-minimum consecutive states constraint [16]. In this work, we will also add information about sequence patterns found in the data by using a CRF.

When modeling the activities with an HMM, it is difficult to incorporate prior knowledge in the form of sequence patterns because HMMs have difficulty in modeling overlapping, non-independent features [17]. A Conditional Random Field can be thought of as a more general Hidden Markov Model. With CRFs it is possible to add arbitrary features even if they overlap. We identified recurrent patterns that occur within each activity and coded them as features in CRFs.

In this paper we present an extension of our previous work about long-term activities segmentation [16] with the following additions: (1) the use of real data collected with a wristwatch from two subjects (21 days of data) instead of using simulated data; (2) the use of Conditional Random Fields in order to include more information into the models by finding sequence patterns; (3) the data and code were made publicly available to facilitate the reproduction of the results; (4) the idea of subclassing was introduced in order to deal with the problem of intra-class fragmentation. We also make use of clustering quality measures [18] in order to automatically find good subclassing groups. This problem arises during the activity labeling process. For example, a user could label an activity as *having dinner* but sometimes he may use cutlery (for meat, soups, salads, *etc.*) and sometimes he may use his hands (for hamburgers, pizza, *etc.*). In this case, the activity label is the same for both scenarios even though both may vary widely from the acceleration point of view, making the activity *having dinner* implicitly fragmented into two classes. This phenomenon can introduce noise to the model and decrease the recognition accuracy. In Section 7 we will describe the process to deal with the intra-class fragmentation problem by automatically finding possibly fragmented classes and *subclassing* them.

This paper is organized as follows. Section 2 presents related works on activity recognition. Section 3 presents the background theory of Hidden Markov Models and Conditional Random Fields. In Section 4 we present the data collection process and the preprocessing steps. Section 5 details how the sequences of primitives are generated from the raw accelerometer data. Section 6 explains the features used for the CRF, which includes finding the sequence patterns. Section 7 describes the intra-class fragmentation problem and the proposed method to reduce its effects. In Section 8 we describe the experiments and present the results. Section 9 directs to the sources for downloading the data and code in order to reproduce the results. Finally, in Section 10 we draw conclusions and propose future work.

## 2. Related Work

Recent works have taken advantage of wearable sensors to perform simple activity recognition with them. Many of these works use motion sensors (e.g., accelerometer and gyroscope) to recognize simple physical activities like running, walking, sleeping, cycling, among other activities [19–24]. To perform simple activity recognition, usually, the accelerometer data is divided into fixed time windows, generally 2–10 s [19,23,25]. Then, time domain and/or frequency domain features [26] are extracted from the data. The set of features of the corresponding window is known as a feature vector or an instance. Those feature vectors are used to train and test classification models such as Decision Trees, Naïve Bayes, Support Vector Machines, and other types of classifiers [27].

There has also been research on complex and *long-term activities*. Martínez-Pérez *et al.*, implemented a system in a nursing home [1]. Their system integrates artifact behavior modeling, event interpretation and context extraction. They perform the inference based on how people handle different types of artifacts (devices to measure blood pressure, paper towel, cream, *etc.*). In their use case, they characterized the caregivers' activities of taking blood pressure, feeding, hygiene and medication of an elderly patient with restricted mobility over a period of ten days, achieving an accuracy of 91.35%. Gu *et al.* [28] focused on recognizing sequential, interleaved and concurrent activities like making coffee, ironing, drinking, using phone, watching TV, *etc*. They conducted their experiments in a smart home using sensors like accelerometers, temperature, humidity, light, *etc*. They also attached RFID tags to rooms, cups, teaspoons, and books. Their reported overall accuracy was 88.11%. One of the recent works in complex activity recognition is that of Cook *et al.* [29]. In their work, they do activity discovery and activity recognition with data collected from three smart apartments with several installed sensors and they deal with the interesting problem of having unlabeled activities. Each apartment housed an elderly during six months. They used infrared motion detectors and magnetic door sensors to recognize 11 different activities like bathing, cooking, eating, relaxing, *etc*. They reported accuracies of 71.08%, 59.76% and 84.89% for each of the three apartments.

There are also works that do complex activity recognition using wearable sensors. In the work of Huynh *et al.* [30], they used three wearable sensors boards: one on the right wrist, one on the right side of the hip and the last one on the right thigh. The sensor boards have 2D accelerometers and binary tilt switches. They recorded three different high level activities (housework, morning tasks and shopping) from one user giving a total of 621 min. They tested different algorithms and achieved the best accuracy (91.8%) when using histogram features with a Support Vector Machine. Mitchell *et al.* used accelerometers from smartphones to classify sporting activities: five matches of soccer (15 players) and six hockey matches (17 players) [31]. They achieved a maximum F-measure accuracy of 87% by the fusion of different classifiers and extracting features using the Discrete Wavelet Transform. One of the advantages of using wearable sensors for activity recognition is that it is easy to uniquely identify the users. When using sensors installed in an environment with multiple residents, it becomes difficult to identify which user activated a specific sensor. Another advantage with wearable sensors is that the recognition can be performed almost in any place. A disadvantage of using a wearable sensor, e.g., just one accelerometer, is that it is not possible to detect activities that do not involve the movement of the part of the body that has the sensor. For example, if the accelerometer is in the user's pocket and he is sitting down, it may not be possible to tell whether he is working on a computer or maybe having dinner. Installed sensors and wearable sensors are complementary approaches to perform human activity recognition, both with strengths and weaknesses. For example, in [32] the authors used a multi-sensor approach with wearable (accelerometer) and installed sensors (cameras).

When recognizing simple activities, the approach of extracting features from window segments is appropriate since they last for only a few seconds. However, to recognize long-term activities from *accelerometer* data, generating fixed length windows is not suitable. For example, the long-term activity *working* could have small periods of the walking activity, but this does not mean that the user is commuting to another place. If a fixed time window method is used, it may be the case that the window includes only data about the walking activity so the model can confuse this activity with *commuting*. Generally, users perform long-term activities in a continuous manner and they do not have a clear separation boundary. In order to recognize more complex activities from accelerometer data, models that can deal with temporal information are needed. Examples of such models are Hidden Markov Models (HMMs) and Conditional Random Fields (CRFs), which have already been used extensively for activity recognition. For example, Lee and Cho used hierarchical HMMs to recognize activities like taking bus, shopping, walking, among other activities, using data collected with a smartphone [33]. Their method consists of first recognizing actions using HMMs and then using these actions sequences to feed higher level HMMs that perform the activity recognition. In [34], the authors used HMMs to recognize actions from several inertial sensors placed in different parts of the body. They used nine sensor boards in different parts of the body and their experiments included three subjects. In the previously mentioned work [29], Cook *et al.* also used a CRF and an HMM. On the other hand, Van Kasteren *et al.* used HMMs and CRFs to monitor activities for elderly care from data collected from a wireless sensor network in a smart home [35]. In the work of Vinh *et al.*, they used semi-Markov CRFs, which enabled them to model the duration and the interdependency of activities (dinner, commuting, lunch, office) [36]. Their reported precision was 88.47% on the dataset from [37], which consists of data collected during seven days using two triaxial accelerometers. Table 1 summarizes some of the related works for complex activity recognition.

This work differs from the previous ones in the following aspects. (1) We use a single accelerometer in a wristwatch to perform long-term activity segmentation; (2) We propose a method to deal with the problem of intra-class fragmentation by finding possible subclasses of each class by means of clustering quality indices. We found that the overall recognition accuracy was improved (within our tested approaches) by incorporating prior knowledge such as the activities minimum lifespan and by finding sequence patterns that are common to each of the activities. *Subclassing* fragmented activities also helped to increase the overall recognition accuracy within our tested approaches.

## 3. Background

In this section we introduce the notations that will be used throughout this paper, as well as the background theory of Hidden Markov Models and Conditional Random Fields. From here on, we will use the notation introduced by Rabiner and Juang ([10], Chapter 6).

### 3.1. Hidden Markov Models

A Hidden Markov Model is a probabilistic graphical model consisting of a set of observations and states represented by random variables and can be defined as a 5-tuple 〈*N*, *M*, *A*, *B*, *π*〉 where:

- *N* is the number of states in the model indexed by {1, 2, …, *N*}. The current state at time *t* is *q_t_*.
- *M* is the number of distinct observation symbols denoted as *V* = {**v**_1_, **v**_2_, …, **v***_M_*}.
- *A* is the state transition probability distribution. *A* = {*a_ij_*} where
  (1) $$\begin{array}{ll} a_{ij}=P(q_{t+1}=j|q_{t}=i), & 1\leq i,j\leq N \end{array}$$
  and *a_ij_* ≥ 0 ∀*j*, *i*; $\begin{array}{ll} \overset{N}{\underset{j=1}{\text{∑}}}a_{ij}=1 & \forall i \end{array}$
- *B* is the observation symbol probability distribution. *B* = {*b_j_*(*k*)} in which
  (2) $$\begin{array}{ll} b_{j}(k)=P(o_{t}=v_{k}|q_{t}=j), & 1\leq k\leq M, \end{array}$$
  defines the symbol distribution in state *j*,*j* = 1, 2, …, *N*.
- *π* is the initial state distribution *π* = {*π_i_*} in which
  (3) $$\begin{array}{ll} \pi_{i}=P(q_{1}=i), & 1\leq i\leq N. \end{array}$$

### 3.2. Conditional Random Fields

**Figure 1.**
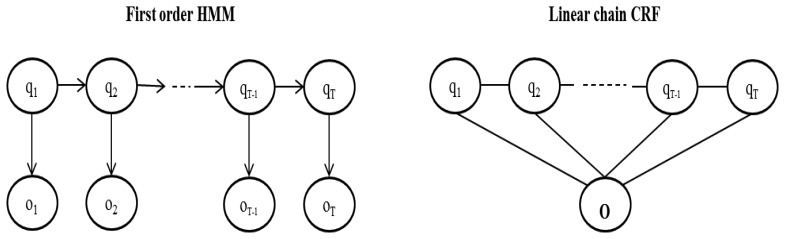
A visual comparison between a first order HMM and a linear chain CRF. The HMM defines a joint probability P(**O**, **Q**) whereas the CRF defines a conditional probability P(**Q** | **O**). Note that an HMM only has access to the current observation **o***_t_* but the CRF has access to the entire observation sequence **O** at any given time.

Hidden Markov Models are generative models that define a joint probability distribution *P*(**O**, **Q**) where **O** and **Q** are random variables. An observation **o***_t_* may only depend on the state at time *t* [41] (Figure 1). This is a strong assumption—in real world scenarios an observation may depend also on states at different times. For example, in part-of-speech tagging [42] the aim is to label a sequence of words with tags like “PERSON”, “COMPANY”, “VERB”, *etc*. For example, if “*Martin Inc.* …” is the sequence of words, as soon as we see “*Martin*” we may label it as “PERSON” but if we look at the next observation “*Inc.*” then we realize that the first tag should be “COMPANY”. This type of dependencies can be easily modeled with a CRF.

Conditional Random Fields were first introduced by Lafferty *et al.* [15] to label sequenced data. Linear chain Conditional Random Fields define a conditional probability:

(4) $$P(Q|O)=\frac{1}{Z}\exp\left(\sum_{t=1}^{T}\sum_{i=1}^{F}\omega_{i}f_{i}(q_{t-1},q_{t},O,t)\right)$$

where **Q** is the states sequence and **O** is the observations sequence, *f_i_* and *ω_i_* are feature functions and their respective weights and can take real values. *F* is the total number of feature functions.

Since the feature functions can take arbitrary real values, the *Z* term is used as a normalization factor to make it a valid probability and is defined as the sum of exponential number of sequences:

(5) $$Z=\sum_{Q}\exp\left(\sum_{t=1}^{T}\sum_{i=1}^{F}\omega_{i}f_{i}(q_{t-1},q_{t},O,t)\right).$$

The feature functions take as arguments the previous and current state *q_t_*_−1_, *q_t_*, the entire observations sequence **O** and the current position *t*. Note that, unlike HMM that only have access to the current observation at any given time, CRF have access to the entire observations sequence. An example of a feature function may be:

(6) $$f_{i}(q_{t-1},q_{t},O,t)=\{\begin{array}{ll} 1 & \text{if}\;q_{t}=\text{COMPANY and}\\ & O_{t+1}=\text{Inc}.\\ 0 & \text{otherwise}. \end{array}$$

This will have the effect of increasing the probability of state sequences that assign the tag COMPANY to an observation that is followed by the “*Inc.*” word (if the corresponding weight *ω*_1_ is positive). In this case it returns a binary value but it could be any real value.

The main advantage of a discriminative model is that it can easily include overlapping features like in the COMPANY example. Interdependent features can also be included in generative models by enhancing the models or making simplifying independence assumptions. The first approach becomes difficult when trying to retain tractability. Making simplifying assumptions can be done and works well for some applications but may degrade the performance for some others [17]. For linear CRFs (which are the ones used in this work) inference algorithms can be performed efficiently by variants of the standard dynamic programming methods for HMMs. For general graphs, inference problems become intractable and approximate methods are needed. In general, computing the normalization factor *Z* requires exponential time; however, since for this application we want to know what is the most likely sequence of states regardless of the exact probability value, we can omit that computation.

To perform the activity inference, we used the Viterbi algorithm on both HMMs and CRFs. The constrained version of the Viterbi algorithm [16] allows to set the minimum number of consecutive states that can occur for each state *i*. This requires an additional parameter *k*, which is an array that stores the minimum consecutive states for each activity:

(7) $$\begin{array}{ll} \kappa (i)=n\in {\mathbb{N}}_{>0}, & 1\leq i\leq N \end{array}$$

Now we can take into account the minimum lifespan of each activity by coding this information in the *κ* array This information was computed from the data by finding the minimum number of consecutive states of each activity and dividing them by 2 (to allow some deviance).

## 4. Data Collection

**Figure 2.**
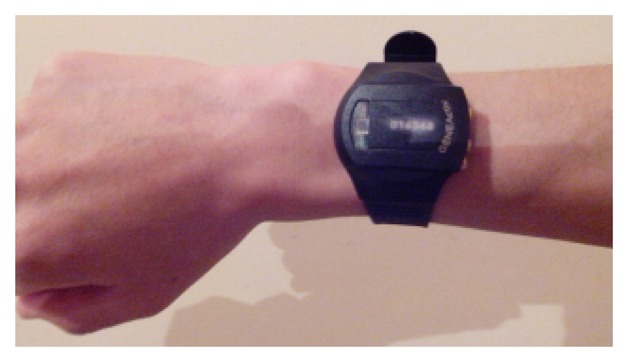
Device used to collect the data.

**Figure 3.**
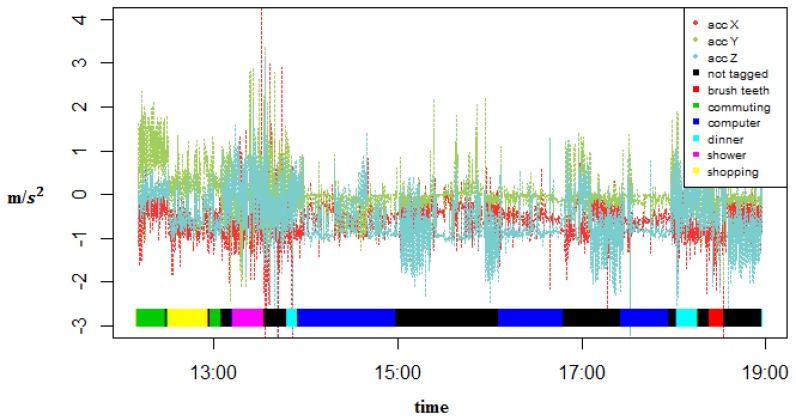
Raw accelerometer data and tagged activities during one specific day.

A GENEActiv [43] watch with an embedded triaxial accelerometer was used to collect the data. This watch can sample at up to 100 Hz. For our experiments, the sampling rate was set at 20 Hz because it has been shown that activity classification is high for sampling rates above or equal to 10 Hz but the accuracy decreases at a sampling rate of 5 Hz [44]. This watch also collects temperature and light intensity but they were not used. The watch was placed on the dominant hand of each user (Figure 2). The users performed their activities of daily living and took note of the start and end time of several of them. Data were collected by two subjects during 11 and 10 days, respectively, from approximately 9:00 to 23:00. The first subject was one of the researchers and the second subject was a volunteer that had no relation to this work. The first subject recorded 6 activities: (1) *Shopping*, which consists of picking and buying groceries at the supermarket; (2) *Showering*, which consists of taking a shower and dressing; (3) *Dinner*, which also takes into account breakfast and lunch time; (4) *Working*, which for most of the time is working with a computer but also involves taking rests and going to the bathroom; (5) *Commuting*, which includes by bus, by car, or walking; (6) *Brush Teeth*, which consists of both brushing the teeth and using a dental floss. Subject 2 tagged 4 activities: (1) *Commuting*; (2) *Lunch*, which also consists of breakfast and dinner time; (3) *Working time*, which for most of the time is office work; (4) *Exercise*, which consists of walking, running, stretching, *etc*. There is also an activity *not tagged* for data that was not labeled by the users. Figure 3 shows the raw accelerometer data and the tagged activities for one specific day of one of the subjects. Tables 2 and 3 show the number of instances and the duration of each activity for both subjects.

To reduce some noise, an average filter with a window length of 10 (Equation (8)) was used:

(8) $$v_{s}(t)=\frac{1}{n}\sum_{i=t-n}^{t-1}v(i)$$

where *v* is the original vector, *v_s_* is the smoothed vector and *n* is the window length.

## 5. Sequence of Primitives Generation

**Figure 4.**
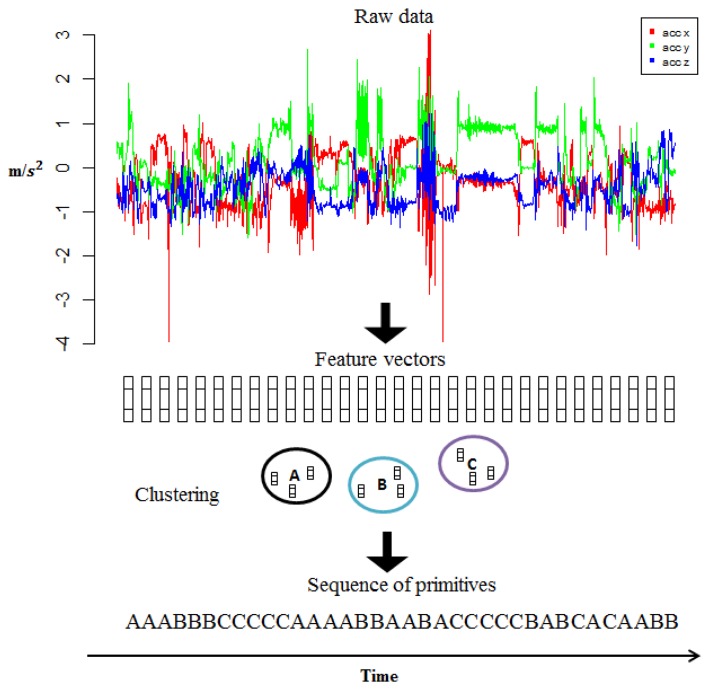
The acceleration signals are transformed into sequences of primitives using vector quantization.

A long-term activity is characterized by a sequence of primitives. These primitives are automatically discovered from the data. In the context of HMMs and CRFs they will be the observations. To obtain the sequence of primitives, the data is divided into windows of fixed length *l* (we used *l* = 60, which is ≈ 3 s) with an overlap of 33%, because in [25] it was shown that small window sizes lead to better results than using longer window sizes. For each window segment, the next set of 12 features were computed: mean and variance for each of the *x,y* and *z* axes; the correlation between every pair of axes; and the mean, variance and average derivative of the magnitude. The resulting feature vectors do not have a label or class. The task consists of categorizing the feature vectors into different groups such that instances that are very similar to each other belong to the same group. For this step we used the *k*-means clustering method, where *k* represents the number of clusters we want. Next, the center or the centroid of each cluster will correspond to a primitive with a unique ID. This clustering step is just performed on the training set. To assign a label/class to an instance, we get the ID of the closest centroid and assign it as the label of the instance. Figure 4 depicts the overall process of vector quantization.

## 6. CRF Feature Functions and Training

In this section we describe each of the three feature functions that were included in the model and how they were estimated from the training data.

### 6.1. *f*_1_ Observation Symbol Probability Distribution

This feature function is equivalent to Equation (2) for HMMs and defines the probability of observing an specific symbol (*primitive*) given some state (activity).

(9) $$f_{1}(q_{t-1},q_{t},O,t)=\log(P(o_{t}|q_{t})).$$

These probabilities are estimated from the training data as:

(10) $$P(O_{k}|q)=\frac{\text{number of primitives of class}\;k\;\text{in}\;q}{\text{total number of primitives in}\;q},\begin{array}{l} 1\leq q\leq N\\ 1\leq k\leq M \end{array}$$

The reason for the log() function is to make this feature equivalent to the symbol probability distribution of an HMM.

### 6.2. *f*_2_ State Transition Probability Distribution

This is equivalent to the state transition probability distribution of an HMM (Equation 1).

(11) $$f_{2}(q_{t-1},q_{t},O,t)=\{\begin{array}{ll} \log(\pi_{qt}) & \text{if}\;t=1\\ \log(P(q_{t}|q_{t-1})) & \text{otherwise} \end{array}$$

where *π_i_* are the initial probabilities that were set to be uniform. The probability of transitioning from one state to another *P*(*q_t_* ∣ *q_t_*_−1_) is estimated by computing the transition frequency for every pair of states. Generally, the probability of transitioning from a state to itself will be much higher than transitioning to another state.

### 6.3. *f*_3_ Sequence Patterns (k-Mers)

The purpose of this function is to include information about the sequence structure, *i.e.*, to find overlapping sequences of primitives that occur together. In bioinformatics a *k*-*mer* (also called *n*-grams, *n*-mers, *l*-tuples) ([45], p. 308) is a string of length *k* and *Counti*(*Text*, *Pattern*) is the number of times a *k*-*mer Pattern* appears as a substring of *Text.* For example:

Co«nt(ACA**CTAC**TGCATA**CTACTAC**CT, **CTAC**) = 3 Note that the patterns can be overlapped. Usually, *k*-*mers* are used in DNA sequence analysis [45]. In text processing they are usually called n-grams [46]. The total number of different *k*-*mers* is *M^k^*; recall that M is the number of distinct observation symbols. The feature function *f*_3_ is defined as:

(12) $$f_{3}(q_{t-1},q_{t},O,t)=\log(P(o_{t},\ldots ,o_{t+k-1}|q_{t}))$$

which is the probability of finding a specific *k*-*mer* given an activity. This feature function is based on one of the implications of Zipf's law and is described in [46] in the context of document classification as: “if we are comparing documents from the same category they should have similar N-gram frequency distributions”. Note that this feature function is similar to *f*_2_ when *k* = 2 but instead of activity transitions, it defines *primitives* transitions, *i.e.*, the probability of some primitive *v_i_* occurring after some primitive *v_j_* given an activity. In the training phase we estimate these probabilities as:

(13) $$(P(o_{t},\ldots ,o_{t+k-1}|q)=\frac{\text{Count}(\text{PrimSe}q_{i},k-\text{mer})}{|\text{PrimSe}q_{i}|-k+1},1\leq i\leq N$$

for all *M^k^* possible *k*-*mers*. *PrimSeq_i_* is the concatenation of the *primitives* sequence for all long-term activities of type *i* from the training set.

## 7. Subclassing Fragmented Classes

One of the problems when tagging the activities in the training phase is the intra-class fragmentation. This means that several observations may belong to the same class even though they are completely different. For example, a user may tag an activity as *commuting* but sometimes he may commute mainly by bus and other times by walking, which are very different from the acceleration point of view. This will produce noise in the models since the training will take into account all observations of the same class in conjunction, but in the recognition phase an observation may belong to just one subclass (commuting by bus or walking). Of course the user could create two different classes at training time (bus commute and walk commute) but this would be a tedious task and the subclasses may not be obvious. What we propose is the following sequence of steps:

(1) For each class *C_i_* in the training set, find if it is fragmented.
(2) If it is fragmented, assign each of its observations to their corresponding subclass *C_i,j_* for *j* = 1…F where F is the total number of fragments.
(3) If it is not fragmented, assign all observations to a single subclass *C_i_*_,1_.
(4) Build/train the models with the subclasses as if they were independent classes.
(5) Predict the subclass of the observations in the test set.
(6) For the test set, replace each predicted subclass *C_i,j_* with the original class *C_i_*.
(7) Validate the results with the ground truth (original class labels).

**Figure 5.**
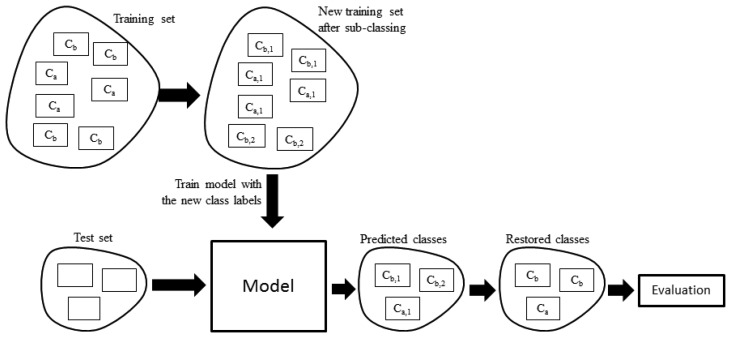
Overall process for performing the subclassing.

**Figure 6.**
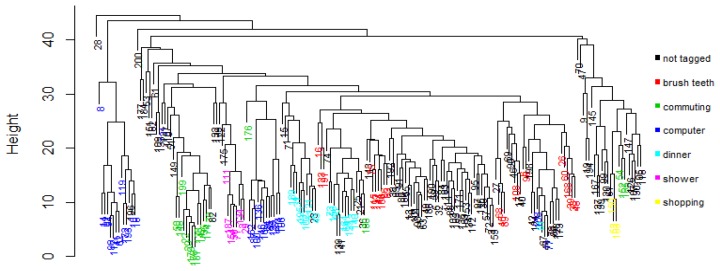
Resulting dendrogram of hierarchical clustering for subject 1.

Figure 5 shows the overall process. One way to find if a class is fragmented is by visual inspection. One approach to perform the visual inspection is to cluster the observations using a method that does not require specifying the number of groups a priori, e.g., using hierarchical clustering and then plotting the resulting dendrogram. Figure 6 shows the resulting dendrogram when applying hierarchical clustering to the observations of subject 1. From this dendrogram, we can see that the *working with computer* class is fragmented roughly into two, the *shopping* activity looks uniform, the *commuting* activity looks like it is fragmented into two, *etc*. In some cases it is not obvious in how many fragments a class is divided into. For example, the *brush teeth* activity could be divided into three or maybe four subclasses so this approach is subjective and may just work well when the number of classes is small and the separation of the fragments is more or less clear.

To perform the clustering, each long-term activity is represented as a sequence of *primitives* and will be characterized with a feature vector of size M, where M is the total number of different *primitives*, *i.e.*, the alphabet size. The feature vector stores the frequencies of each of the *primitives* so each long-term activity is characterized by a distribution of *primitives* (a *histogram*).

The visual approach for finding the fragments of a class is feasible when (1) we deal with a small set of activities, and (2) when we have predefined knowledge about the subclasses, *i.e.*, an expert is able to validate from human experience. In the case of external validity, that is, when the user knows the class labels or the number of subclasses, good cluster structure is accomplished when the predicted classes are the same as the predefined labels of the dataset. When either of the two characteristics mentioned before is not fulfilled, a more flexible approach must be used. In the absence of prior knowledge, the use of relative validity metrics [47] may offer a good approximation to the real segmentation.

Let us recall that the objective of partitioning techniques is to cluster groups of elements that are highly related in terms of similarity. As a consequence, the idea is to find clusters where the elements of each group are as close to each other as possible (*et al.*, compactness), and the distance between the clusters is widely spaced (*et al.*, separation). In this case, cluster structure is measured in terms of clustering quality indices [18], which indicate the quality of cluster structure based on compactness and separation. Some relative validity indices widely used include Dunn Index, Davies-Bouldin Index, Silhouette Index, Maulik-Bandoypadhyay Index, PBM Index [47–49].

In this work we used the Silhouette [50], PBM [49] and Generalized Dunn's Index (GDI33) [51] clustering quality indices. Silhouette and GDI33 were chosen because in [18] they proved to work well with different datasets. PBM was chosen because it is more recent and in the original paper [49] it was claimed that the results were superior than other previously proposed indices.

The following process to get the optimal number of fragments with respect to some quality measure is based on the steps described in [52] and was applied just to the training set:

(1) Cross-validate the training set.
(2) For each class *C_i_*, cluster the observations that belong to *C_i_* into *k* groups, for *k* = 2…*maxK*. *maxK* is a predefined upper bound on the allowed number of fragments. Compute the quality index of the clustering result for each *k*.
(3) Subclass *C_i_* into *k* subclasses where *k* is the number of groups that yielded the optimal quality index.
(4) Cross-validate the training set with the new subclasses.
(5) If the resulting accuracy improves the result of step 1, keep the new subclassing for *C_i_*.

For our experiments, in the cross-validation step, instead of building the HMMs and CRFs, we used the Naive Bayes classifier with the activities *histograms* as input. The reason of validating the subclassing step with a simpler classifier is because it is much faster than building the HMMs and CRF models and this step has to be done *maxK* times for each class. For the clustering step, we used the *k*-means algorithm. One of the advantages of finding the fragments using a quality index is that it can be done automatically without user intervention as opposed to the visual approach described earlier. It is worth noting that the discovered sub-activities may not correspond to “real activities” since the clustering process is based in kinematic features and, as a consequence, activities are split according to their qualities rather than their real sub-type. However, for the purposes of this work this is not relevant since the subclassing is exploited to increase the overall accuracy rather than used to understand what these sub-activities are.

## 8. Experiments and Results

Recall that a long-term activity is represented as a sequence of *primitives*. A run will be defined as a concatenation of long-term activities that occur consecutively, *et al.*, an entire day. We performed five experiments: (1) without subclassing; (2) with fixed subclassing; (3) with silhouette subclassing (4) with PBM subclassing; (5) with GDI33 subclassing. Fixed subclassing means that the number of subclasses for each class were set manually by visual inspection of the dendrogram. In each of the experiments we compared three different methods to perform the activity segmentation:

(1) **HMM**: Viterbi algorithm on an HMM without constraint.
(2) **HMM** + **C**: Viterbi algorithm on an HMM with the k-minimum-consecutive-states constraint.
(3) **CRF**: Viterbi algorithm on a CRF with the k-minimum-consecutive-states constraint and adding information about sequence patterns.

**Figure 7.**
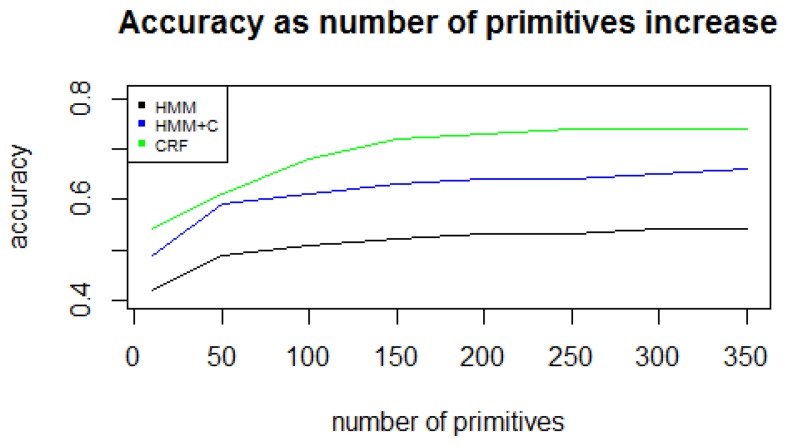
Accuracy as number of primitives increase.

Since long-term activities are user-dependent, *et al.*, for one person, the *working* activity may involve doing office work and for another person the *working* activity may be more physically demanding, individual models with the same parameters were built for each subject. Leave-one-day-out cross validation was used to validate the results. This means that for each day *i*, all other days *j*, *j* ≠ *i* are used as training set and day i is used as the test set. The accuracy was computed as the percentage of correct state predictions. Due to computational limitations, the *k* for *k-mers* was set to be a small number (in this case, 2) because the number of *k-mers* grows exponentially (*M^k^* = 300^2^ = 90, 000 possible different patterns). The number of *primitives* was set to 300 because the accuracy does not have a significant increase for values greater than 250 (Figure 7). The number of primitives parameter depends on the sensor data and the features extracted. In our previous work [9] we collected data from a cellphone located on the user's belt and used a different set of features. In that case, a parameter value greater or equal to 15 gave the best results. In [11] they used a MotionNode sensor to detect simple activities. In that case, an alphabet size of around 150 gave the best results. The number of primitives will depend on each application and is determined empirically as stated in [11]. The overall results for each of the five experiments are listed in Table 4.

From Table 4 it can be seen that for the five experiments the accuracy increases as more information is added to the model—first adding the minimum activities lifespan (HMM + C) and then adding both the minimum lifespan and the sequence patterns (CRF). To assess the influence of the transitions between activities, instead of learning the transition matrix from the data we set it to have a uniform distribution between any two possible transitions (to represent a lack of information). In this case, the overall accuracy (no subclassing) was 52.0%, 67.9%, and 75.5% for HMM, HMM + C and CRF, respectively. This suggests that the transitions between activities had a considerable influence in the first model (HMM), but HMM + C and CRF were more robust to the choice of the transition matrix. The type of activities considered in this work may have strong predictable diurnal patterns. As a baseline, we performed the activity recognition with no accelerometer data by just using information of the time of the day. First, the predicted activity at a given time was set as the activity with highest probability of occurring at that specific time of the day based on the training data. This resulted in an overall accuracy of 44%. Then, we used the Viterbi algorithm on an HMM with the time of the day as observations, resulting in an overall accuracy of 47%. These results suggest that just using accelerometer data, long-term activities can be detected independently of the time of the day and with higher accuracy than relying on just time daily patterns. This is especially true for *high-entropy subjects*, which tend to be more variable and do not follow a fixed schedule and are thus harder to predict based on their daily routines patterns [53].

**Figure 8.**
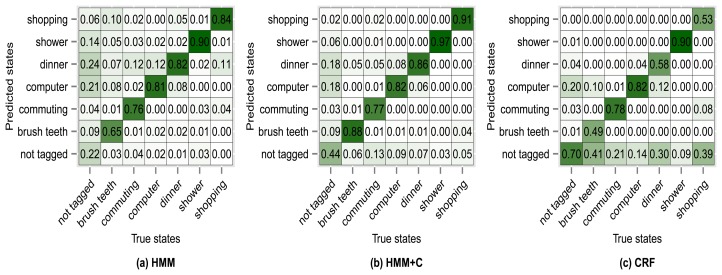
Confusion matrices for experiment 1 (subject 1): no subclassing.

Figure 8 shows the confusion matrix of experiment 1 for subject 1. From Figure 8a we can see that 76% of the observations that were generated by the *commuting* activity were correctly classified as being generated by the *commuting* activity. We can also see that 2% of the observations that were generated by the *commuting* activity were misclassified as if they had been generated by the *shopping* activity. The antidiagonal of the matrices shows the sensitivity (true positive rate). For example *brush teeth* has a sensitivity of 0.65, commuting has a sensitivity of 0.76, *etc*. One thing to note is that the sensitivity tends to increase for all activities when using HMM + C with respect to HMM. When using a CRF, the sensitivity for the *not tagged* states increases significantly but for some of the activities it drastically decreases. Even though the overall accuracy when using a CRF increases, the sensitivity of some activities decreases. It seems that using HMM + C is a good choice for a tradeoff between overall accuracy and activity sensitivity. One of the reasons that increasing the sensitivity of *not tagged* states and decreasing the sensitivity of the other activities yield an overall higher accuracy is because most of the states are of type *not tagged* (Tables 5 and 6). Almost half of the data was not tagged by the users. Tagging the activities is tedious for a user and consumes time. One possible approach that we are going to explore for future work is to fill missing tags using information from other users. The idea is not to force the user to continuously tag the data but to let the system automatically fill missing information obtained from other sources (a crowdsourcing approach).

We used a paired Student's *t*-test to statistically validate whether or not there is a significant difference when performing the recognition without subclassing and with silhouette subclassing. This was done for each of the three algorithms: HMM, HMM + C and CRF. The null hypothesis is that μ_0_ − μ*_s_* = 0 (the mean accuracy when not subclassing μ_0_ is the same as when using silhouette subclassing μ*_s_*). The alternative hypothesis is that μ*_s_* − μ_0_ > 0, *et al.*, there is a significant increase in the overall accuracy when using silhouette subclassing. The significance level for the tests was set at *α* = 0.05.

**Figure 9.**
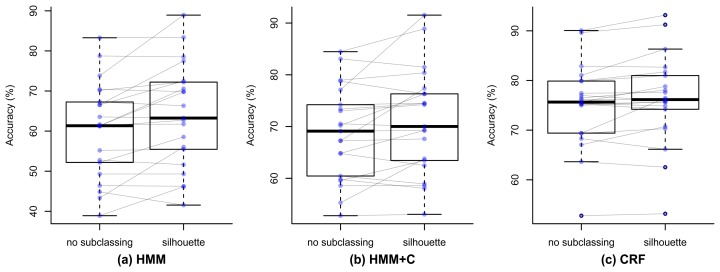
Paired box plots showing the accuracies for the 21 days.

Figure 9 shows the paired box plots that represent the accuracy for each of the 21 days. It can be seen how the mean accuracy increases when performing the silhouette subclassing for the three algorithms. Table 7 shows the results of the formal statistical tests to validate this. The second column presents the difference of the overall accuracy when not subclassing and when using silhouette subclassing. The *t*-test assumes that the data is normally distributed, so we used the Shapiro–Wilk normality test [54] to validate this assumption and the resulting *p*-values are shown in the third column. The fourth column shows the resulting p-value of the paired *t*-test. The null hypothesis of the Shapiro–Wilk test is that the data has normal distribution. Since the *p*-value for HMM and HMM + C is smaller than the significance value 0.05, we can conclude that the data is not normal, which violates the assumption. The *t*-test for this case can still be done but the results should be taken with caution. The *t*-test *p*-values for the three algorithms are smaller than the significance level 0.05, so we can conclude that there is a significant increase in the accuracy when using silhouette. Since the normal assumption was violated for the CRF case, we also validated the results with a Mann–Whitney U test, which is non-parametric and does not assume a normal distribution of the variables. The results of these tests are shown in the last column and all *p*-values also resulted significant.

**Figure 10.**
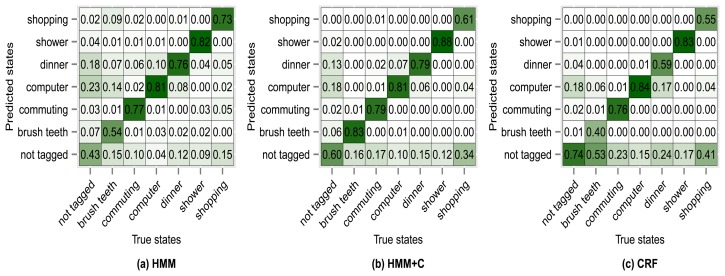
Confusion matrices for experiment 2 (subject 1): fixed subclassing.

**Figure 11.**
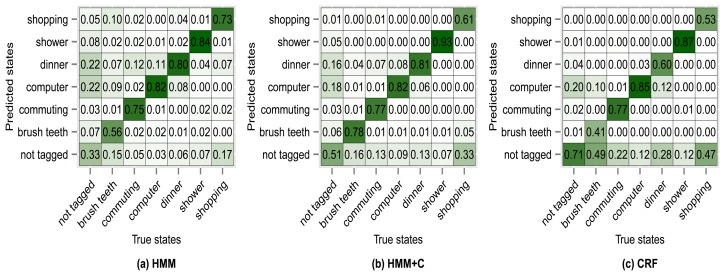
Confusion matrices for experiment 3 (subject 1): silhouette subclassing.

**Figure 12.**
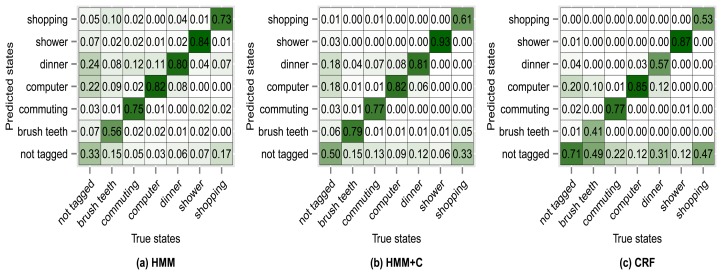
Confusion matrices for experiment 4 (subject 1): PBM subclassing.

**Figure 13.**
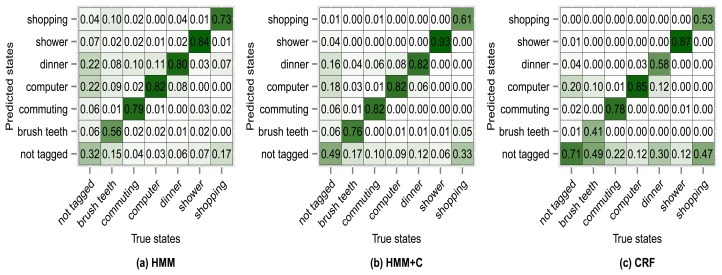
Confusion matrices for experiment 5 (subject 1): GDI33 subclassing.

The results of using other clustering quality indices (PBM, GDI33) produced very similar confusion matrices and overall accuracies compared with using the silhouette index. Higher accuracies were obtained when applying a fixed subclassing by visually inspecting the resulting dendrogram, but the difference is small compared with the use of clustering indices. Figures 10, 11, 12 and 13 show the confusion matrices for experiments 2,3,4 and 5 for subject 1.

## 9. Reproducibility

In order to make the results reproducible, we made the data and code available for download [55,56]. The implementation was coded in R and Java programming languages. The code uses the HMM R package [57] and a modified version of its Viterbi function in order to include the *k*-minimum-consecutive states constraint. Both the data and the code files include a description of the data and the instructions to run the code.

## 10. Conclusions

In this work, we performed long-term activity segmentation from accelerometer data collected with a wristwatch. The long-term activities were transformed into sequences of simple activities by using vector quantization. HMMs and CRFs were used to perform the segmentation. It was shown how adding additional information to the models helped to increase the overall accuracy of the tested approaches. The additional information consisted of the minimum lifespan of each of the activities and sequence patterns (*k*-*mers*). Most of the works on complex activity recognition use a fixed infrastructure of sensors. There are also works that perform the recognition using different types of wearable sensors as described in Section 2. In this work, we explored the use of a single accelerometer to segment different long-term activities, which may be used as an indicator of how independent a person is and as a source of information to healthcare intervention applications. Most of the works also assume that a complex activity has always the same distribution of simple activities that compose it, which may not be the case since the same conceptual activity can be performed in very different ways. To deal with this issue, we introduced the idea of subclassing and described how to reduce the impact of intra-class fragmentation by subclassing the activities using visual inspection and clustering quality indices. The most similar works are [30,36], which achieved an accuracy of 91.8% and a precision of 88.47%, respectively. The former collected 10 h of data from one user and the latter used a dataset consisting of 7 days of data from one user. In this work, we achieved accuracies between 70.0% and 77% when using subclassing with HMM + C and CRFs for two different users (total 21 days of data) and 7 different activities using just one sensor. One of the limitations is that both users reported similar activities and in both cases the *working* activity involved office work, thus further evaluation with a wider range of users and activities is still needed to test the generalization of the method to other possible scenarios and types of users. Another limitation is that the difference between simple and long-term activities was explained to the volunteer subject before the data collection process, hence only long-term activities were tagged. As a consequence, the system assumes that the tagged data consist of just long-term activities but in real world scenarios the users should be allowed to personalize an activity recognition system with any type of activity regardless of its type. In addition, further evaluation needs to be done to see how well this approach could be applied to detecting activities in real time rather than doing the computations offline. This is important in order to provide direct support to individuals while the activity is taking place rather than waiting after the data has been collected.

In our experiments, most of the data was not labeled by the user, which can cause several problems. For example, the user may not have labeled a known activity *i* but at test time it may be correctly classified as *i*. Since it does not initially have a label, it will be marked as misclassified. To overcome this, for future work we will use a crowdsourcing approach to complete missing information. The idea of using crowdsourcing for activity recognition from video data is already being explored [58,59]. However, for accelerometer data it presents several challenges because it is hard to classify an activity based on visual inspection.

## Figures and Tables

**Table 1.** Related complex activity recognition works.

**Work**	**# Activities**	**Sensors**	**Details**
Martínez-Pérez *et al.* [1]	4: taking blood pressure, feeding, hygiene, medication	RFID, accelerometers, video cameras	91.35% accuracy, 1 patient during 10 days. 81 instances.
Gu *et al.* [28]	26: making coffee, ironing, using phone, washing clothes, *etc*.	accelerometers, temperature, humidity, light, RFID, *etc*.	Overall accuracy 88.11%, 4 subjects over a 4 weeks period. Collected instances 532.
Cook *et al.* [29]	11: bathing, cooking, sleeping, eating, relaxing, taking medicine, hygiene, *etc*.	infrared motion detectors and magnetic door sensors	Accuracies of 71.08%, 59.76% and 84.89% for each of the 3 apartments during a period of 6 months.
Huynh *et al.* [30]	3: housework, morning tasks and shopping.	2D accelerometers and tilt switches	Accuracy of 91.8% for 1 user and period of about 10 h.
Kasteren *et al.* [35]	bathing, dressing, toileting, *etc*.	reed switches, pressure mats, mercury contacts, passive infrared, float sensors and temperature sensors	4 different datasets
Tolstikov *et al.* [38]	7: leaving, toileting, showering, sleeping, breakfast, *etc*.	14 binary sensors	Maximum accuracy of 95.7% for 1 subject during 27 days.
Vinh *et al.* [36]	4: dinner, commuting, lunch and office work	2 triaxial accelerometers	Precision of 88.47% for data collected during 7 days.
Sung *et al.* [39]	12: cooking, talking on the phone, working on computer, *etc*.	Microsoft Kinect	Average precision 86.5%, data collected by 4 subjects
Gordon *et al.* [40]	7: drinking, gesticulating, put mug on table, meeting, presentation, coffee break, *etc*.	accelerometers attached to mugs	Average accuracy of 95% for single-user and maximum 96% for group activities. 3 subjects. In total over 45 mins. of collected data.

**Table 2.** Number of instances and duration of each self-reported activity (subject 1).

	**Brush Teeth**	**Commuting**	**Computer**	**Dinner**	**Not Tagged**	**Shopping**	**Shower**
**# Instances**	23	23	31	20	109	3	10
**Total hours**	2.9	9.4	32.4	5.0	50.1	1.0	3.5

**Table 3.** Number of instances and duration of each self-reported activity (subject 2).

	**Commuting**	**Lunch**	**Work**	**Exercise**	**Not Tagged**
**# Instances**	54	17	21	3	95
**Total hours**	14.6	6.4	38.3	1.0	51.7

**Table 4.** Overall accuracies for the five experiments for the two subjects.

	**HMM**	**HMM+C**	**CRF**
**Experiment 1: no subclassing**	60.7%	68.8%	75.1%
**Experiment 2: fixed**	64.7%	71.9%	77.1%
**Experiment 3: silhouette**	64.1%	71.0%	76.3%
**Experiment 4: PBM**	63.9%	70.8%	76.2%
**Experiment 5: GDI33**	64.1%	71.0%	76.3%

**Table 5.** Percent of states of each class (subject 1).

	**Brush Teeth**	**Commuting**	**Computer**	**Dinner**	**Not Tagged**	**Shopping**	**Shower**
**(%)**	2.8	9.0	30.9	4.8	47.9	1.0	3.4

**Table 6.** Percent of states of each class (subject 2).

	**Commuting**	**Exercise**	**Lunch**	**Not Tagged**	**Work**
**(%)**	13.1	0.9	5.8	46.1	33.8

**Table 7.** Resulting *p*-values of the statistical tests (*μ*_0_: mean accuracy with no subclassing, *μ_s_*: mean accuracy with silhouette subclassing).

**Algorithm**	***μ*_0_ − *μ****_s_*	**Shapiro–Wilk *p*-Value**	***t*-Test *p*-Value**	**Mann–Whitney *p*-Value**
**HMM**	−3.5	*p* ≪ 0.05	*p* ≪ 0.05	*p* ≪ 0.05
**HMM+C**	−2.2	*p* ≪ 0.05	*p* ≪ 0.05	*p* ≪ 0.05
**CRF**	−1.1	*p* ≫ 0.05	*p* ≪ 0.05	*p* ≪ 0.05
